# Development of Keyword Trend Prediction Models for Obesity Before and After the COVID-19 Pandemic Using RNN and LSTM: Analyzing the News Big Data of South Korea

**DOI:** 10.3389/fpubh.2022.894266

**Published:** 2022-04-29

**Authors:** Gayeong Eom, Haewon Byeon

**Affiliations:** ^1^Department of Statistics, Inje University Graduate School, Gimhae, South Korea; ^2^Department of Digital Anti-Aging Healthcare (BK21), Graduate School of Inje University, Gimhae, South Korea; ^3^Department of Medical Big Data, College of AI Convergence, Inje University, Gimhae, South Korea

**Keywords:** obesity, COVID-19 pandemic, text mining, topic modeling analysis, LSTM

## Abstract

The Korea National Health and Nutrition Examination Survey (2020) reported that the prevalence of obesity (≥19 years old) was 31.4% in 2011, but it increased to 33.8% in 2019 and 38.3% in 2020, which confirmed that it increased rapidly after the outbreak of COVID-19. Obesity increases not only the risk of infection with COVID-19 but also severity and fatality rate after being infected with COVID-19 compared to people with normal weight or underweight. Therefore, identifying the difference in potential factors for obesity before and after the pandemic is an important issue in health science. This study identified the keywords and topics that were formed before and after the COVID-19 pandemic in the South Korean society and how they had been changing by conducting a web crawling of South Korea's news big data using “obesity” as a keyword. This study also developed models for predicting timing before and after the COVID-19 pandemic using keywords. Topic modeling results was found that the trend of keywords was different between before the COVID-19 pandemic and after the COVID-19 pandemic: topics such as “degenerative arthritis”, “diet,” and “side effects of diet treatment” were derived before the COVID-19 pandemic, while topics such as “COVID blues” and “relationship between dietary behavior and disease” were confirmed after the COVID-19 pandemic. This study also showed that both RNN and LSTM had high accuracy (over 97%), but the accuracy of the RNN model (98.22%) had higher than that of the LSTM model (97.12%) by 0.24%. Based on the results of this study, it will be necessary to continuously pay attention to the newly added obesity-related factors after the COVID-19 pandemic and to prepare countermeasures at the social level based on the results of this study.

## Introduction

The World Health Organization (WHO) declared the COVID-19 outbreak a global pandemic on March 11, 2020 (1). As COVID-19 has spread afterward, lockdown has been declared worldwide including South Korea, the United States, and countries in Europe and Asia (2). Moreover, prolonged social distancing has changed people's lifestyles (2). Eating out, going out, traveling, and gatherings have decreased, and telecommuting, non-face-to-face classes, and video conferencing have increased, which are typical examples. As a result, more people stay only at home and the radius of activity has decreased, which has decreased physical activities.

From the nutritional science aspect, the representative change is the rapid increase in food delivery and convenience food after the outbreak of COVID-19. The “COVID-19 Impact Report” published in April 2020 revealed that the proportion of delivered meals nearly doubled after the COVID-19 pandemic: from 33 to 52% (3). Moreover, the consumption of processed foods and convenience foods has increased mainly due to convenience and taste rather than nutrition (4, 5). This dietary behavior may increase the risk of diseases such as obesity, diabetes, hypertension, and metabolic syndrome due to excessive intake of saturated fat and an imbalance in essential nutrients (6, 7). Changes in nutritional intake and dietary behavior under the COVID-19 pandemic have made people gain weight (8, 9), which is believed to greatly affect daily life by causing mental and physical problems (10). In other words, the COVID-19 pandemic has created an environment that makes prone to obesity by causing problems, such as a decrease in physical activity, irregular eating, and depression (11).

Due to these changes, the Korea National Health and Nutrition Examination Survey (2020) (12) reported that the prevalence of obesity (≥19 years old) was 31.4% in 2011, but it increased to 33.8% in 2019 and 38.3% in 2020, which confirmed that it increased rapidly after the outbreak of COVID-19. In Korea, people with confirmed COVID-19 infection are called “hwakjinja.” After the COVID-19 pandemic, the obesity rate has risen sharply, and obesity is emerging as a social problem. It is serious enough to create an online coinage–“hwakzinja” (pun)–for those who have gained weight. Particularly, many previous studies (13–15) have reported that obesity increases not only the risk of infection with COVID-19 but also severity and fatality rate after being infected with COVID-19 compared to people with normal weight or underweight. Therefore, identifying the difference in potential factors for obesity before and after the pandemic is an important issue in health science. Although obesity has become a more serious social issue, there are not enough studies using the text mining technique to understand it. The text mining technique, which targets text data accumulated for a long time such as news, is academically more valuable because it can discover social and cultural issues and examine the trends of changes (16, 17). Therefore, it is necessary to uncover the macro trends of language, society, and culture by identifying the increasing or decreasing trend in the frequency of language use and focusing on the correlation between them (16) and carefully examine words if these specific words show a certain usage pattern.

This study identified the keywords and topics that were formed before and after the COVID-19 pandemic in the South Korean society and how they had been changing by conducting a web crawling of South Korea's news big data using “obesity” as a keyword. This study also developed models for predicting the trend of keywords based on RNN and LSTM.

## Materials and Methods

### Data Collection and Analysis Period

This study utilized BIGKinds (www.bigkinds.or.kr), a news archive site of the Korea Press Foundation, to collect data necessary for analysis. Table 1 shows the media analyzed in this study. This study selected 52 media: eleven metropolitan newspapers, 28 local newspapers, eight economic newspapers, and five broadcasting companies.

**Table 1:** The analyzed media.

**Type**	**Number of media**	**Name**
Metropolitan newspaper	11	Kyunghyang Shinmun, Kookmin Ilbo, Naeil Newspaper, Donga Ilbo, Munhwa Ilbo, Seoul Newspaper, Segye Ilbo, JoongAng Ilbo, Chosun Ilbo, Hankyoreh, and Hankook Ilbo
Local newspaper	28	Gangwon Provincial Daily, Gangwon Ilbo, Gyeonggi Ilbo, Gyeongnam Provincial Daily, Gyeongnam Daily, Gyeongsang Ilbo, Gyeongin Ilbo, Gwangju Ilbo, Gwangju Daily Daily, Kukje Daily, Daegu Ilbo, Daejeon Ilbo, Maeil Daily, Mudeung Ilbo, Busan Ilbo, Yeongnam Ilbo, Ulsan Daily, Jeonnam Ilbo, Jeonbuk Provincial Daily, Jeonbuk Ilbo, Jemin Ilbo, Joongdo Ilbo, Jungbu Daily, Jungbu Ilbo, Chungbuk Ilbo, Chungcheong Ilbo, Chungcheong Today, and Halla Ilbo
Economic newspaper	8	Maeil Economy, Money Today, Seoul Economy, Asian Economy, Aju Economy, Financial News, Korea Economy, and Herald Economy
Broadcasting company	5	KBS, MBC, OBS, SBS, and YTN

The analysis period was divided into before and after the COVID-19 pandemic for efficient analysis. Before the COVID-19 pandemic was from February 28, 2019, to March 10, 2020, and after the COVID-19 pandemic was from March 11, 2020, to December 31, 2021. The search word for data collection was “obesity,” and the number of news texts for analysis is presented in Table 2.

**Table 2:** The number of analyzed news texts (number of cases).

	**Before the COVID-19 pandemic**	**After the COVID-19 pandemic**	**Total**
First collected news	5,201	7,217	12,418
Excluded news	1,362	2,226	3,588
Analyzed news	3,839	4,991	8,830

This study initially collected 12,418 text (news) data by using “obesity” as a keyword. Then, this study analyzed 8,830 texts after excluding 3,588 news, which were duplicated or not related to nutrition or health. This study conducted data preprocessing and extracted morpheme using the refined data obtained from BIGKinds.

### Analysis Methods

**Figure 1:**
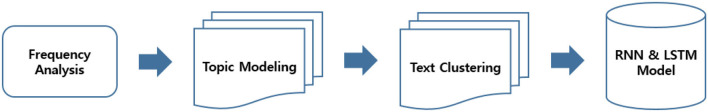
Flowchart of the study.

Figure 1 presents the analysis procedure of the study. First, this study examined the appearance frequency of words in the entire text (whole collected text (news) data) by using frequency analysis. Second, this study identified keywords based on the COVID-19 pandemic period and changes in topics related to “obesity” by using latent Dirichlet allocation (LDA) topic modeling. Third, this study clustered objects (topics) and understood the distribution of clusters based on similarity by using text clustering. Fourth, lastly, this study built models to predict topics by using recurrent neural network (RNN) and long short term memory (LSTM), deep learning algorithms, and compared their predictive performance.

#### Latent Dirichlet Allocation Topic Modeling

**Figure 2:**
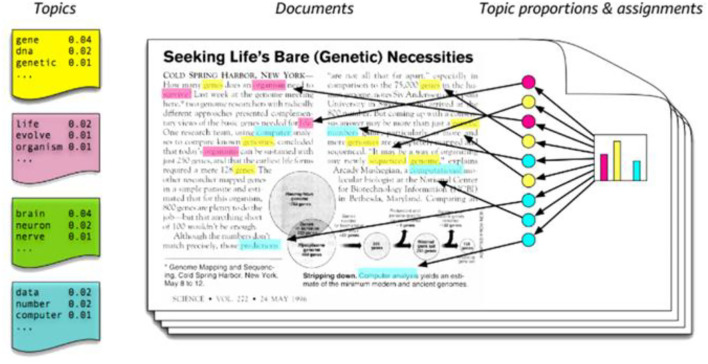
The overview of topic modeling process (19).

LDA is a topic modeling method proposed in 2003, and it is a text mining technique to extract latent core topics from documents or texts in literature (18). Topic modeling compresses the literature expressed by the combination of numerous words into a relatively small number of potential topics and allows us to concisely grasp the contents, which are advantages of this method. In particular, LDA is useful for analyzing potential topics by using text-based big data such as news. Therefore, it is widely applied to various natural language processing. The concept of the LDA algorithm is presented in Figure 2.

#### Design of LDA Topic Modeling

**Figure 3:**
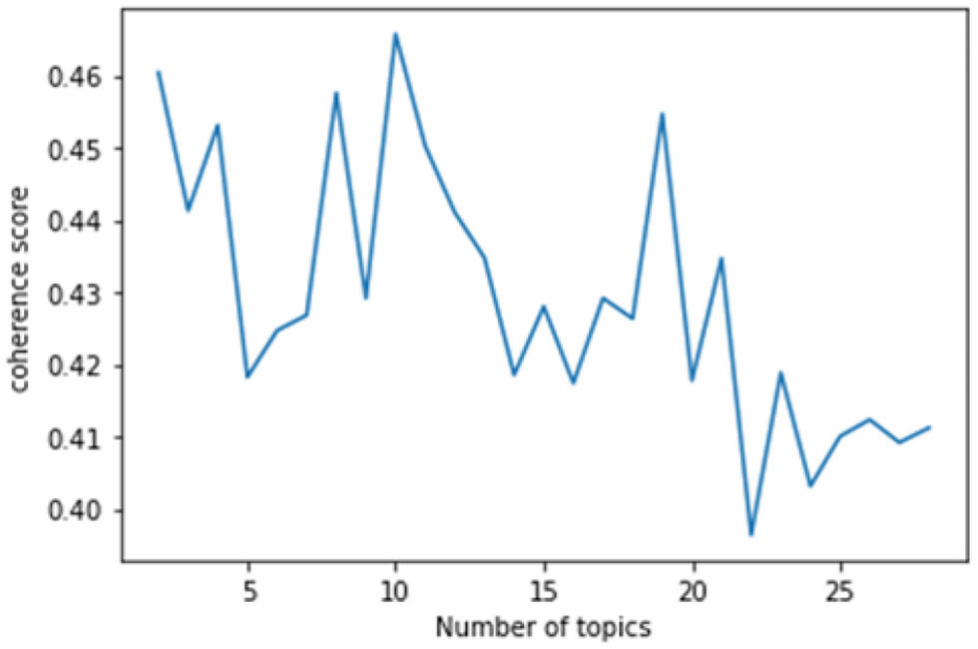
Estimated coherence scores (before the COVID-19 pandemic).

**Figure 4:**
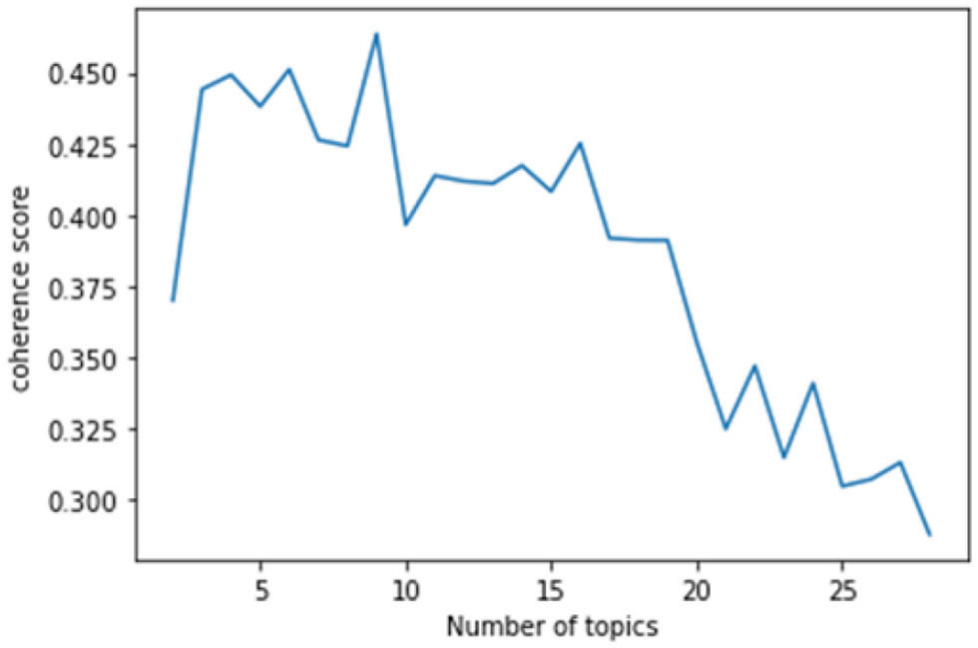
Estimated coherence scores (after the COVID-19 pandemic).

This study used the genism module (Python) to conduct LDA topic modeling. This study visualized the topic modeling by using the coherence model of the gensim module and generated a coherence score graph according to the number of topics. The coherence score is a metric used to determine the number of topics, and it numerically calculates whether the words in each topic semantically agree with the number of given topics (x-axis). Therefore, the number of topics with the highest coherence score is the optimal number of topics to the target data of the topic modeling. The coherence score of this study indicated that there were ten topics before the COVID-19 pandemic (Figure 3) and eight topics after the COVID-19 pandemic (Figure 4).

#### K-Means Clustering Algorithm

Cluster analysis is a data mining technique that groups similar data together. Here, a cluster refers to a data group with similar characteristics, and the k-means cluster algorithm groups the given data into k clusters. It works in a way that minimizes within-cluster variances. Unlike supervised learning with a pre-determined label, k-means clustering does not have a standard label. Therefore, the k-means clustering algorithm automatically configures categories based on the variance and distance of feature vectors based on the data. In the k-means algorithm, all data must belong to the closest cluster. It randomly selects k centroids corresponding to the specified number of clusters. Afterward, when a new vector is added, new vectors are allocated to preselected k centroids, and centroids are updated again.

#### Recurrent Neural Network

**Figure 5:**
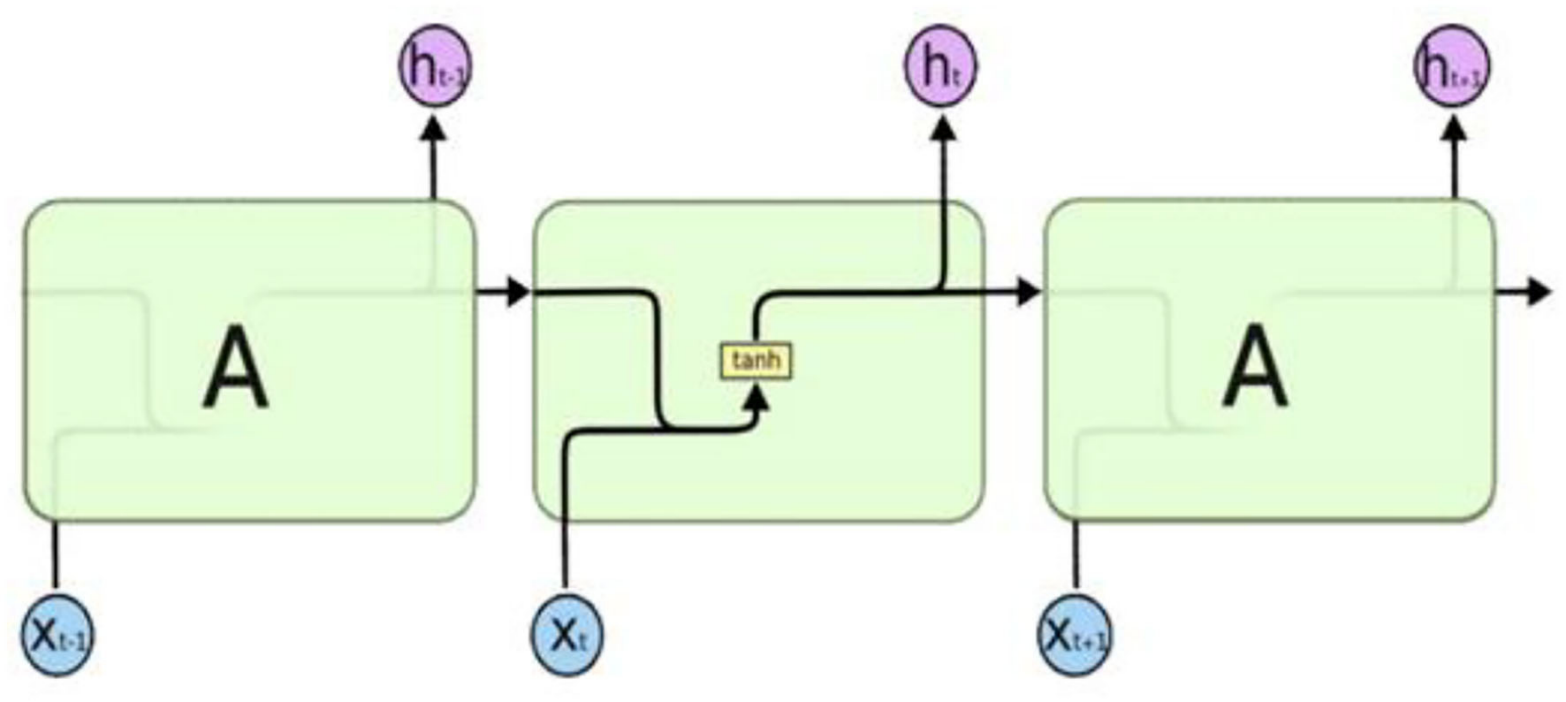
Recurrent neural network structure.

The recurrent neural network (RNN) is a deep neural network model specialized in processing sequential (ordered) information like natural language (20). RNN is an artificial neural network with a circulation structure, rather than flowing signals in one direction, which is a characteristic of it, and the output autoregressively refers to past output data. RNN is mainly used for processing natural language or translation because its structure is appropriate for them. In other words, words in natural language (verbal output) data often need to be understood according to the context rather than the lexical meaning. As a result, RNN is often used to overcome issues in natural language processing. The RNN structure is as follows, and its schematic is shown in Figure 5 (21).

#### Long Short-Term Memory

**Figure 6:**
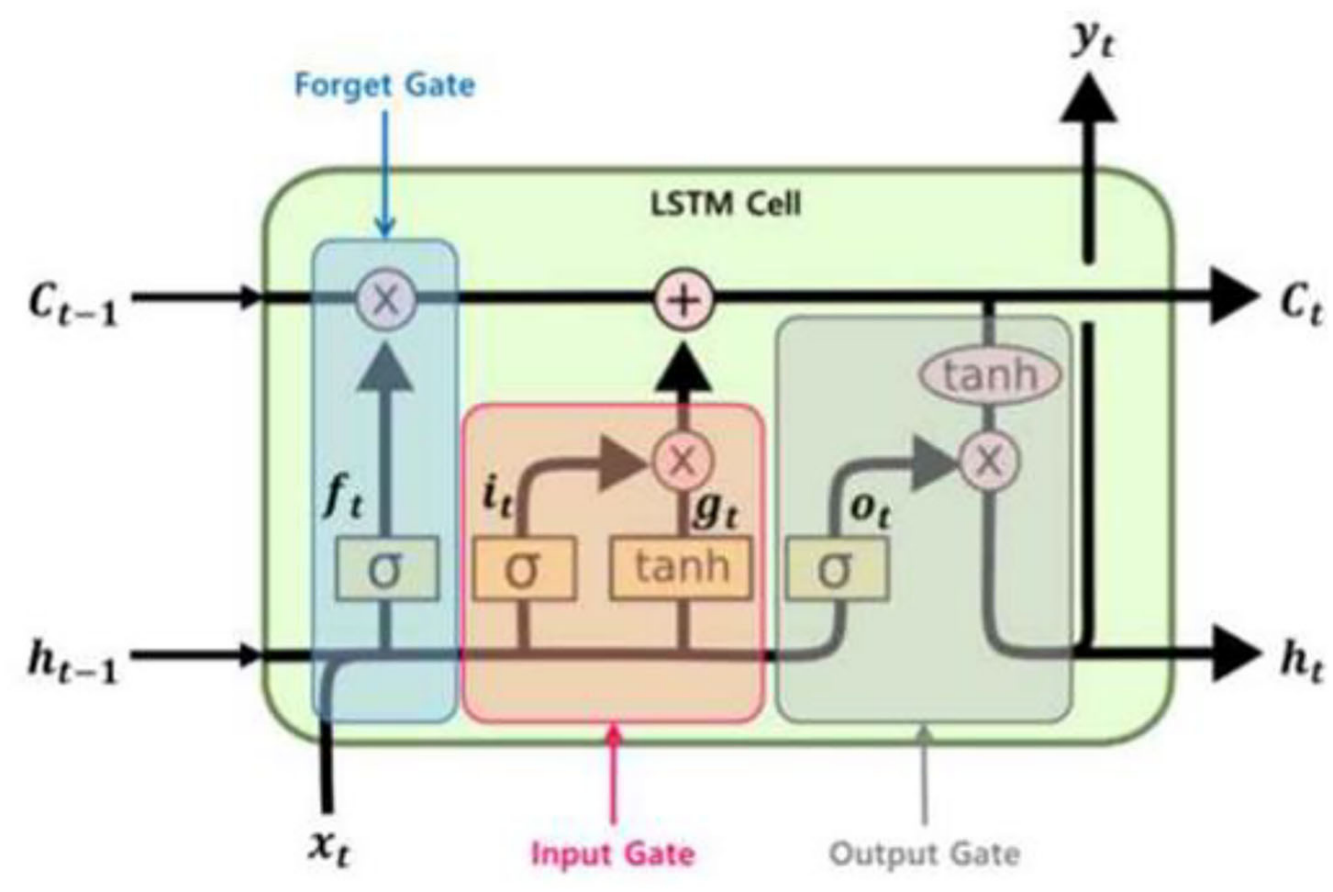
Structure of long-short term memory.

LSTM is a method designed to resolve the gradient vanishing, gradient exploding, and long-term dependency problems of RNN. LSTM has the characteristics of RNN, but it is structured to allow the user to confirm the contents to be remembered in short-term and long-term conditions by dividing the state into short-term and long-term, unlike the RNN cell. Moreover, it can delete some information by using the forget gate, which is different from RNN. Due to these characteristics, LSTM is able to resolve the problems of the conventional RNN and process data more effectively. Figure 6 shows the schematic diagram of LSTM's structure (21).

#### Method of Building RNN Models and LSTM Models

This study divided the data into a training dataset and a validation (experiment) dataset in an 8:2 ratio. The RNN model and LSTM model in this study were binary classification models that labeled “before the COVID-19 pandemic” as 0 and the “after the COVID-19 pandemic” as 1. Therefore, this study used the binary cross entropy as a loss function and the sigmoid function as an activation function. Moreover, this study optimized hyperparameters such as the L2 regulation and the dropout rate to prevent overfitting in the learning process. Table 3 shows the results of the hyperparameter exploration for RNN and LSTM models in this study.

**Table 3:** Exploration results of hyperparameters.

	**RNN**	**LSTM**
Layer	2	2
Timesteps	12	12
Hidden node	64	32
L2	0	0
Dropout rate	0	0.1
Batch size	32	20
Epoch	7	10

## Results

### Frequency Analysis

This study conducted frequency analysis on the keywords of news texts published before the COVID-19 pandemic and showed that the frequencies of keywords related to “health” were high (Table 4). Moreover, many keywords related to obesity-related diseases (e.g., hypertension, diabetes, hyperlipidemia, metabolic syndrome, cardiovascular disease, chronic disease, complication, and adult disease) were derived. In addition, keywords related to healthcare (e.g., dietary behavior, dietary life, protein, nutrient, healthcare, physical activity, lifestyle, prevention, program, exercise, operation, and obesity prevention) were derived.

**Table 4:** Top 30 frequencies before the COVID-19 pandemic.

**Word**	**Frequency**	**Word**	**Frequency**	**Word**	**Frequency**
Health	580	Physical Activity	240	Cardiovascular Disease	174
Public health center	521	Lifestyle	232	Operation	168
Hypertension	467	Protein	218	Body Mass Index	158
Diabetes	452	Prevention	197	Chronic Disease	158
Obesity	409	Body Weight	189	Nutrient	158
Dietary behavior	391	Hyperlipidemia	187	Complication	153
Dietary life	324	Program	187	Overweight	149
Healthcare	305	Possibility	185	BMI	148
Obesity ratio	284	Metabolic Syndrome	183	Obesity Prevention	144
Onset of a disease	262	Exercise	180	Adult Disease	138

This study carried out frequency analysis on the keywords of news published after the COVID-19 pandemic and keywords such as “COVID-19,” “amount of activity,” “online,” “depression,” “immunity,” and “confirmed case” were added (Table 5).

**Table 5:** Top 30 frequencies after the COVID-19 pandemic.

**Word**	**Frequency**	**Word**	**Frequency**	**Word**	**Frequency**
COVID-19	2,179	Obesity	311	Overweight	199
Diabetes	655	Cardiovascular Disease	299	Body Weight	187
Hypertension	570	Protein	291	Lifestyle	187
Public health center	440	Complication	265	Depression	177
Health	379	Hyperlipidemia	229	Fatty Liver	172
Onset of a disease	365	Dietary Life	214	Immunity	172
Healthcare	346	Metabolic Syndrome	214	Confirmed Case	171
Dietary behavior	343	Amount of Activity	212	Prevalence	161
Physical activity	321	Online	202	Nutrient	161
Possibility	320	Obesity Rate	199	BMI	156

### LDA Topic Modeling

Table 6 presents the results of LDA topic modeling using news articles published before the COVID-19 pandemic. This study presented top five words per topic because the weight of a topic drastically dropped from the sixth-ranked word. The first topic was “degenerative arthritis” and consisted of “possibility,” “arthritis,” “onset of a disease,” “gene,” and “lifestyle.” It was found that topic #2 (“diet”) and topic #10 (“side effects of diet treatment”) were regarding diet. It was also found that topics #6 (“metabolic syndrome”), #8 (“cardiovascular disease”), and #9 (“chronic disease”) were diseases caused by obesity.

**Table 6:** Topic modeling before the COVID-19 pandemic.

**Topic**	**Topic name**	**Top 5 words in each topic**
1	Degenerative arthritis	Possibility, arthritis, onset of a disease, gene, and lifestyle
2	Diet	Obesity, body weight, health, diet, and fat
3	Health promotion project (obesity Prevention Program)	Health, obesity, prevention, program, and public health center
4	Health promotion project (physical activity promotion)	Public health center, obesity rate, healthcare, physical activity, and operation
5	Protein intake	Protein, nutrient, onset of a disease, intake, and muscle mass
6	Relationship between metabolic syndrome and body weight	Metabolic syndrome, body mass index, overweight, BMI, and body weight
7	Nutrition education	Dietary behavior, dietary life, nutrition education, nutritionist, and obesity rate
8	Cardiovascular disease	Korean, cardiovascular disease, belly fat, dietary therapy, and family medicine
9	Chronic disease	Diabetes, hypertension, hyperlipidemia, complications, and chronic diseases
10	Side effects of diet treatment	Side effects, treatment, medicine, medical staff, and weight loss

Table 7 shows the results of LDA topic modeling using news articles published after the COVID-19 pandemic. The difference from the topics before the COVID-19 pandemic was the appearance of topics related to COVID-19, such as topics #3 (“COVID blues”) and #4 (“COVID-19 treatment”). It was also confirmed that “smartphone,” a new keyword, was added to topic #5 (“health promotion project”), which was different from the before the COVID-19 pandemic. This is presumed to be due to changes in health care methods because of the transition to the contactless era due to the COVID-19 pandemic. It is also believed that topic #8 (“protein intake”) was added because protein (a nutrient for improving immunity) intake has become important to prevent infectious diseases such as COVID-19.

**Table 7:** Topic modeling after the COVID-19 pandemic.

**Topic**	**Topic name**	**Top 5 words in each topic**
1	Chronic disease	Diabetes, hypertension, cardiovascular disease, onset of a disease, and gene
2	Obesity diagnosis	Overweight, BMI, body mass index, waist circumference, and belly fat
3	COVID blues	Onset of a disease, depression, practice rate, amount of activity, and melancholy
4	COVID-19 treatment	COVID-19, treatment, side effects, confirmed case, and infectious disease
5	Health promotion project	COVID-19, public health center, health care, physical activity, and smartphone
6	Relationship between dietary behavior and disease	Dietary behavior, body weight, lifestyle, fatty liver, and hyperlipidemia
7	Prevention and management of obesity	Health, obesity, prevention, program, and exercise
8	Protein intake	Protein, nutrient, possibility, immunity, and aerobic metabolism

### K-Means Clustering

**Figure 7:**
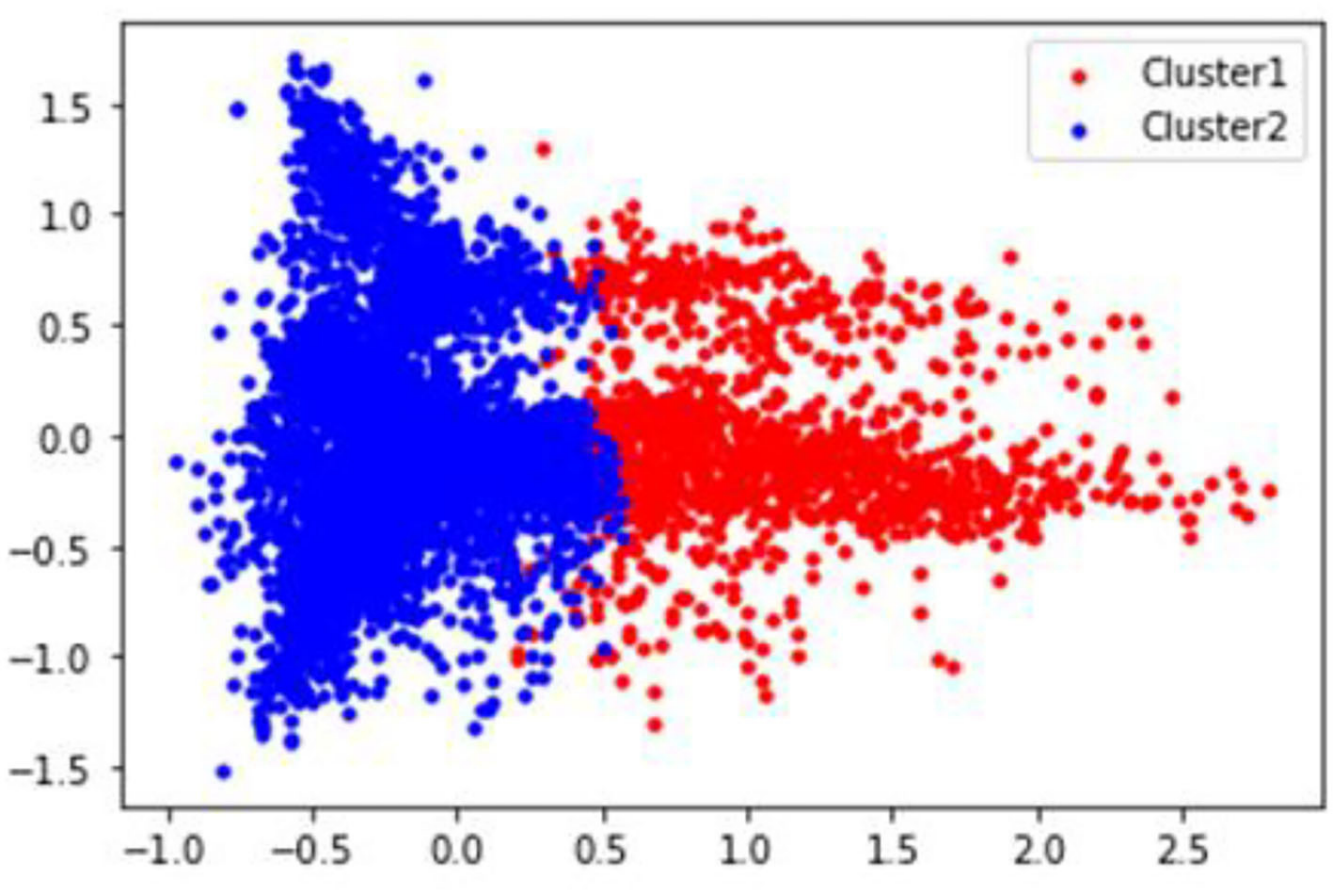
The result of k-means clustering.

This study performed k-means clustering to identify the distribution of news articles based on the distance function before constructing the deep learning-based obesity prediction keyword model. The given data were clustered based on the COVID-19 pandemic (Figure 7) and the boundary between the two clusters was clearly identified.

### Development of Deep Learning-Based Obesity Predictive Model and Validation of Prediction Performance

**Figure 8:**
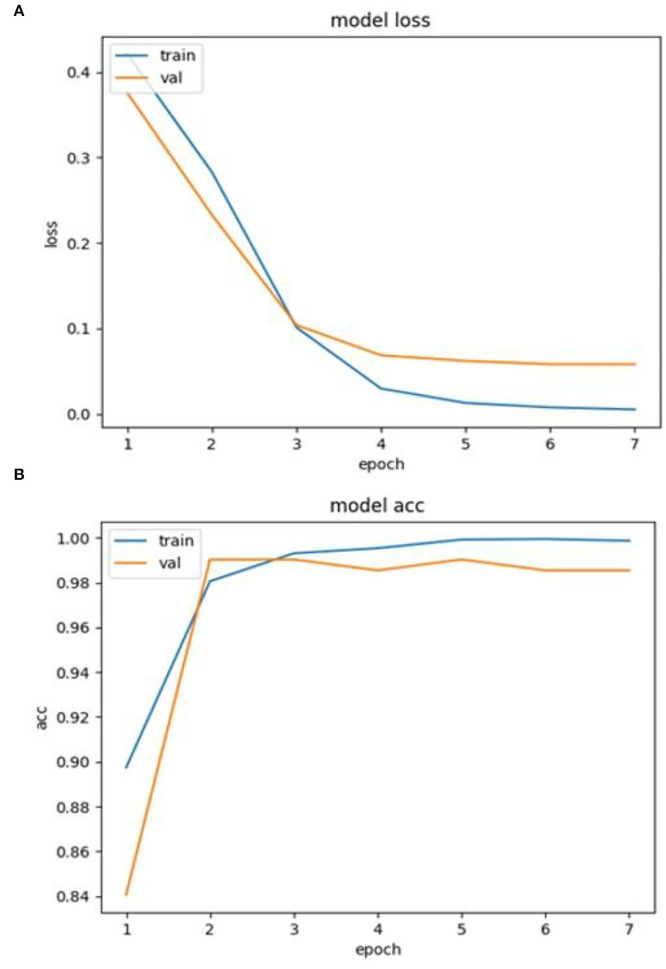
**(A)** Loss and **(B)** accuracy of RNN model.

This study developed RNN or LSTM-based predictive models and compared the predictive performance of them (Figure 8, Table 8). The data were not imbalanced (3,839 data were labeled as 0 and 4,991 data were labeled as 1 out of 8,830 data in total). This study used accuracy, precision, and recall commonly used for examining the predictive performance of the model. The results showed that both RNN and LSTM had high accuracy (over 97%), but the accuracy of the RNN model (98.218%) had higher than that of the LSTM model (97.122%) by 1.096%. Figure 8 depict the loss and accuracy, respectively, of the RNN that showed the best performance in this study. Since the validation loss showed a low decrease from epoch 5, the number of learning was limited to 7 (Figure 8). Moreover, since the validation loss was not overfitted, the RNN model of this study was proven to have excellent performance. In addition, it was confirmed that the RNN-based obesity prediction NLP model of this study had excellent performance because no outliers were detected while the loss decreased sharply and its final accuracy was high (98.218%).

**Table 8:** Predictive performance evaluation (%).

	**RNN**	**LSTM**
Accuracy	98.218	97.122
Precision	97.456	96.717
Recall	89.757	89.669

## Discussion

This study conducted topic modeling using news big data related to obesity. It was found that the trend of keywords was different between before the COVID-19 pandemic and after the COVID-19 pandemic: topics such as “degenerative arthritis,” “diet,” “health promotion project,” and “side effects of diet treatment” were derived before the COVID-19 pandemic, while topics such as “chronic disease,” “obesity diagnosis,” “COVID blues,” “relationship between dietary behavior and disease,” and “protein intake” were confirmed after the COVID-19 pandemic. In particular, coinages appeared after the COVID-19 pandemic, and these new words include COVID-19 blues, where blues means depression (22, 23). It is presumed that the large-scale new infectious disease caused emotional problems such as depression and anxiety (22, 23). Stress affects eating patterns and can lead to overeating or skipping meals (24). Moreover, overeating or skipping due to stress is highly likely to have a serious adverse impact on health by ultimately leading to obesity or weight loss (25). The results of this study implied that emotional disorders such as depression would very highly likely affect not only mental health but also physical health such as obesity after the COVID-19 pandemic. Nevertheless, multiple epidemiological studies (26–28) on community populations after the COVID-19 pandemic have mainly focused on mental health (e.g., depression or anxiety) due to lockdown. More evidence-based epidemiological studies are required to identify changes in physical health such as obesity and related factors after COVID-19 for the community.

Another important finding of this study was that the meaning of a topic could be interpreted differently depending on the period. “Health promotion project” and “protein intake” were confirmed both before and after the COVID-19 pandemic, but their meanings could be different. For example, in this study, COVID-19 and smartphones were identified as the keywords of “health promotion project (Topic #5)” after the COVID-19 pandemic. The trend could be because physical activities decreased after the COVID-19 pandemic due to lockdown and other factors, while the usage time of mobile devices such as smartphones increased (29). Therefore, health promotion projects after the COVID-19 pandemic will need new approaches and systematic prevention and management for health issues that were emphasized less before the COVID-19 pandemic such as smartphone dependency as well as the promotion of physical activity and prevention of obesity.

The results of this study confirmed that the RNN model (98.218%) had higher accuracy than the LSTM model (97.122%) in predicting obesity before and after the COVID-19 pandemic. Contrary to the results of this study, the LSTM model showed better performance than the RNN model in natural language processing using time series data (30, 31). Moreover, Kim et al. (32) analyzed sentiment based on machine learning and showed that LSTM (~89.6%) had better performance than RNN (~84.7%), which was different from this study.

This result could be due to RNN's tendency of losing some information at the beginning of long sentences due to the limitation of gradient vanishing, whereas LSTM has fewer gradient vanishing issues due to its algorithm characteristics. In other words, this study used the feature extraction method that focused on the frequency of keyword occurrence in news articles. The feature extraction does not change the meaning much even if the order of words is changed because it is composed of words, unlike sentence structure. Therefore, when learning using deep learning based on the text using feature extraction, there was no need to consider the previous context of the word. It is believed that the accuracy of the RNN model and that of the LSTM model, which was supplemented with gradient vanishing were not much in this study as a result of that. However, since the prediction performance of the two models was very high (over 98%) in this study, it could not be concluded that RNN was superior to LSTM. Further studies are needed to identify the best natural language processing modeling using various data.

The limitations of this study are as follows. First, the results of this study cannot be generalized to other races or cultures because this study analyzed only the news data of South Korea. As obesity is a global health issue and has been reported as a fatal cause that can increase an individual's chronic and potential disease risk (33), it is needed to conduct more studies on natural language processing to predict obesity by using big news datasets of other cultures for identifying the global trend of obesity after the COVID-19 pandemic. Second, it is not possible to know the trends by age and gender in the changes of the keywords and topics associated with obesity only with the NLP model developed in this study. Although it is known that sociodemographic factors such as gender and age affect obesity (34, 35), this NLP study did not take them into account. Therefore, additional NLP studies are necessary to identify the characteristics of obesity after COVID-19 while considering potential factors influencing obesity such as gender. Third, it is not possible to understand people's perception of obesity only using the NLP study that analyzed news big data. Additional NLP studies on SNS is needed to grasp people's perception of obesity after COVID-19.

Intensive lockdown under the pandemic situation has caused massive social costs (36). It is highly possible that social issues are exacerbated if the health of the local population is deteriorated (e.g., obesity) due to restrictions on social activities (37). The importance of this study was to provide the direction of future health policy by extracting and confirming the keywords and potential topics of obesity from unstructured text-based data before and after the COVID-19 pandemic. It is necessary to develop better natural language processing models by using algorithms with superior performances (38) such as Bidirectional Encoder Representations from Transformers.

## Conclusions

This study evaluated topics related to obesity before and after the COVID-19 pandemic using the news big data of South Korea. This study confirmed topics such as “chronic diseases,” “obesity diagnosis,” “COVID blues” and “relationship between dietary behavior and diseases” and showed that the trend of keywords was different compared to that before the COVID-19 pandemic. This study also developed models for predicting timing before and after the COVID-19 pandemic using keywords. The results revealed that both LSTM and RNN showed high accuracy and the accuracy of RNN was slightly higher. The result implied that it could be more effective to use RNN for news big data using feature extraction. It will be necessary to continuously pay attention to the newly added obesity-related factors after the COVID-19 pandemic and to prepare countermeasures at the social level based on the results of this study. Furthermore, additional NLP studies on SNS are needed to understand the perception of obesity after the COVID-19 pandemic.

## Data Availability Statement

Publicly available datasets were analyzed in this study. This data can be found here:www.bigkinds.or.kr.

## Ethics Statement

The study was conducted according to the guidelines of the Declaration of Helsinki, and approved by the Institutional Review Board (or Ethics Committee) of University (protocol code 20180042 and date: 2018.07.01).

## Author Contributions

GE and HB conceived the study and drafted the manuscript. GE collected the data and performed the statistical analyses. HB critically reviewed and edited the manuscript. All authors contributed to data analysis, drafting and revising the article, gave final approval of the version to be published, have agreed on the journal to which the article has been submitted, and agree to be accountable for all aspects of the work.

## Funding

This research was supported by Basic Science Research Program through the National Research Foundation of Korea (NRF) funded by the Ministry of Education (NRF-2018R1D1A1B07041091 and 2021S1A5A8062526) and 2022 Development of Open-Lab based on 4P in the Southeast Zone.

## Conflict of Interest

The authors declare that the research was conducted in the absence of any commercial or financial relationships that could be construed as a potential conflict of interest.

## Publisher's Note

All claims expressed in this article are solely those of the authors and do not necessarily represent those of their affiliated organizations, or those of the publisher, the editors and the reviewers. Any product that may be evaluated in this article, or claim that may be made by its manufacturer, is not guaranteed or endorsed by the publisher.
